# The Association between Short Periods of Everyday Life Activities and Affective States: A Replication Study Using Ambulatory Assessment

**DOI:** 10.3389/fpsyg.2013.00102

**Published:** 2013-04-15

**Authors:** Thomas Bossmann, Martina Kanning, Susanne Koudela-Hamila, Stefan Hey, Ulrich Ebner-Priemer

**Affiliations:** ^1^Department of Sport and Exercise Science, University of StuttgartStuttgart, Germany; ^2^Department of Sport and Sport Science and House of Competence, Karlsruhe Institute of TechnologyKarlsruhe, Germany; ^3^Department of Psychosomatic Medicine and Psychotherapy, Central Institute of Mental Health, University of HeidelbergMannheim, Germany

**Keywords:** ambulatory assessment, affective states, mood regulation, physical activity, well-being

## Abstract

Regularly conducted exercise programs effectively influence affective states. Studies suggest that this is also true for short bouts of physical activity (PA) of 10 min or less. Accordingly, everyday life activities of short duration might be used to regulate affective states. However, this association has rarely been studied in reference to unstructured activities in ongoing real-life situations. The current study examined the influence of various everyday life activities on three dimensions of mood (valence, calmness, energetic arousal) in a predominantly inactive sample. Ambulatory Assessment (AA) was used to investigate the association between actual PA and affective states during the course of 1 day. Seventy-seven students ages 19–30 participated in the study. PA was assessed with accelerometers, and affective state assessments were conducted hourly using an e-diary with a six-item mood scale that was specially designed for AA. Multilevel analyses indicated that the mood dimensions energetic arousal (*p* = 0.001) and valence (*p* = 0.005) were positively influenced by the intensity of the activity carried out in the 10-min prior to the assessment. As their activity increased, the participants’ positive feelings and energetic arousal increased. However, the students’ calmness was not affected by their activity levels. The findings highlight the importance of integrating short activity intervals of 10 min or less into everyday life routines to improve affective states.

## Introduction

The association between physical activity (PA) and affective states has been of interest for several decades (Arent et al., 2000; Netz et al., 2005). In the field of exercise and psychological adaptations, it is widely acknowledged that structured and organized sports activities have positive effects on subjective well-being (LaFontaine et al., 1992; Reed and Ones, 2006). A meta-analysis carried out by Reed and Ones (2006) verifies this positive effect on various mood parameters (affective states) for low to moderate intensity forms of PA. The meta-analysis found that the effects lasted for at least 30 min and influenced both valence (positive/negative) and energetic arousal (positive activated affect). Greater enhancements were documented when the baseline mood level was lower.

This positive effect on affective states has been shown in several age and patient groups. For example, some studies, focusing especially on young adults (20–30 years old), showed that acute exercise resulted in improved positive affect and less negative affect (Gauvin et al., 1996; Hausenblas et al., 2008; LePage and Crowther, 2010; Kanning et al., 2012). In addition, numerous studies with older and depressive participants have demonstrated the immediate short-term improvements in depressive symptoms and the lasting improvements in affective states that can occur due to various types of PA (Folkins, 1976; LaFontaine et al., 1992; Fox, 1999; Bartholomew et al., 2005; Blake, 2009). Anxious and sedentary people seem to profit most from PA (Folkins, 1976; LaFontaine et al., 1992). Non-depressive persons often exhibit no or minimal effects due to high baseline values of positive affect (LaFontaine et al., 1992). Other studies support the assumption that PA has a more general protective effect on psychological health (Thayer, 1987; Otto and Stemmann, 1991; Gauvin and Spence, 1996; Ekkekakis et al., 2000).

In addition, current research suggests that even short interruptions of sedentary behavior might positively influence health and affective states (Hamilton et al., 2007; Biddle et al., 2010). For example, Thayer (1987) demonstrated that 10 min of brisk walking raised energy levels and reduced tension in adults. The immediate changes lasted for 2 h. Furthermore, Ekkekakis et al. (2000) found that short walking bouts had a positive effect on affective states.

Consequently, it seems that everyday life activities such as walking can be used to improve people’s subjective well-being and psychological health. The likelihood that such forms of PA will be continuously integrated into the everyday life routines of individuals seems higher than the likelihood that individuals will routinely participate in structured, organized physical activities that require more time and effort (Ekkekakis et al., 2000; Schwerdtfeger et al., 2008).

Only few studies have focused on everyday life activities and their effects on affective states (Axelson et al., 2003; Motl et al., 2004; Grossman et al., 2008; Schwerdtfeger et al., 2008; Ebner-Priemer et al., 2008; Powell et al., 2009; Kanning and Schlicht, 2010; Dunton et al., 2011; Kanning et al., 2012). A majority of studies on PA and mood have been conducted in controlled settings such as sports classes or training sessions (for a discussion of this aspect of the research, see Schwerdtfeger et al., 2008; Kanning et al., 2013). In addition, many of these studies have used retrospective questionnaires to assess affective states. Retrospective reports have been criticized as biased due to systematic recall errors (Trull and Ebner-Priemer, 2013).

Unfortunately, definitions of mood, affect, and emotions are quite diverse, and a serious discussion of the differences between these theoretical constructs lies beyond the scope of this paper (for a deeper discussion, see Frijda, 1994; Cabanac, 2002; Scherer, 2009). In the current paper we assume that affective states are volatile and dynamic phenomena (Kuppens et al., 2010). As introduced by several authors (Matthews et al., 1990; Steyer et al., 1997; Schimmack and Grob, 2000), we will base our research on a three-dimensional scale model including *valence* (unwell vs. well), *calmness* (relaxed vs. tense), and *energetic arousal* (tired vs. awake).

Innovative methods are needed to document continuous individual changes in affective states. Unlike research in traditional laboratory settings, such methods assess affective states and PA in real-life situations. Ambulatory assessment (AA) allows the repeated assessment of individual changes in PA levels and affective states over time in everyday life (Bussmann and Ebner-Priemer, 2011; Trull and Ebner-Priemer, 2013). The natural setting and non-invasive procedure provides a less biased assessment of the participants’ affective states. AA has often been called the gold standard in everyday life because it allows (a) the repeated real-time assessment of subjective experiences over long periods of time without retrospective distortions, (b) the real-life assessment of daily activities, and (c) the objective assessment of behavior using accelerometers (Bussmann et al., 2009; Bussmann and Ebner-Priemer, 2011; Trull and Ebner-Priemer, 2013). According to the Society for Ambulatory Assessment (SAA), “*Ambulatory assessment comprises the use of field methods to assess the ongoing behavior, physiology, experience, and environmental aspects of humans or non-human primates in naturalistic or unconstrained settings. Ambulatory assessment designates an ecologically relevant assessment perspective that aims at understanding biopsychosocial processes as they naturally unfold in time and in context*.”^1^

There are still few studies that use AA to investigate the association between unstructured activities and affective states in real time, in real life, and repeatedly over a period of time (see Kanning et al., 2013 for an overview). To our knowledge, only three such studies have been conducted with healthy subjects. Schwerdtfeger et al. (2008) recorded the PA of 124 individuals using accelerometers and assessed mood hourly via handheld computers. They confirmed that there is a significant positive association between PA and energetic arousal and positive activated affect. Significantly, PA is typically a global score that includes intensity and duration of PA. Accordingly, higher intensity and/or duration accompany higher energetic arousal and more positive affect in Schwerdtfeger et al.’s (2008) study. Kanning et al. (2012) investigated the interaction between affective changes, PA, and autonomous regulation in 44 university students using 24-h accelerometry and electronic diaries. Like Schwerdtfeger et al. (2008), the authors reported a positive association between PA and energetic arousal. However, contrary to Schwerdtfeger et al. (2008), they found no significant effect on valence and a significant negative effect on calmness.

Dunton et al. (2011) assessed PA and mood in 121 children using e-diaries and activity monitors but did not report on the relationship between the two variables, as they were interested in context-specific (physical location, social context) interactions. In addition, several studies have assessed the relationship between psychological variables and PA using AA in patients with pediatric affective disorders (Axelson et al., 2003), borderline personality disorder (Ebner-Priemer et al., 2008), and breast cancer (Grossman et al., 2008), as well as those who have just had joint replacement surgery (Powell et al., 2009).

As demonstrated in the above discussion, studies that assess the relationship between everyday life PA and affect on the highest methodological level are rare, and their findings are contradictory. Therefore, the current study aims to clarify the association between PA in everyday life situations and affective states in the course of 1 day. Unlike previous researchers, we examined a sample of students during their exam period; thus, these students are relatively young, fit, and healthy but were inactive at the time of the study. AAs were conducted using electronic diaries and accelerometers to generate objective activity data and avoid retrospective distortions of the affect assessment. Based on recent findings, we hypothesized that the more active people are within the 10-min prior to the affect assessment, the better they feel (valence), the more activated and energetic they feel (energetic arousal), and the less calm they feel (calmness). As Gauvin and Rejeski (2000) indicated that changes in affective states at least partly follow diurnal patterns, we controlled for time of day.

## Materials and Methods

### Participants

The current sample originates from a larger study on workload and stress in students. The original study comprised 149 participants, but PA was assessed in a subsample of 77 students only. We had missing data in 15 subjects, resulting in a final data set with 62 subjects. The participants were between 19 and 30 years old (*M* = 21.4; SD = 1.8); 53 participants were male, and 9 participants were female. The participants’ body mass index varied between 18 and 29 (*M* = 22.1; SD = 2.0). The participants were recruited via a lecture on time management. Accordingly, the sample was entirely composed of students from the Karlsruhe Institute of Technology (KIT, Germany). The study was performed during the exam period at the end of the semester. We assumed that during this period, students spend nearly all of every day at their writing desks preparing for their upcoming exams. Therefore, we expected them to show low PA but felt that this would not indicate that the group is generally inactive. All participants provided informed consent prior to the assessment. The subjects received no monetary compensation for their participation.

### Ambulatory assessment procedure

The study was conducted over the course of 1 week. PA was measured continuously for 24 h using an accelerometer. Electronic diary items were completed via smartphones. The students activated their electronic diaries after waking up, and the measurements were repeated each full hour thereafter. An acoustic signal prompted the participants to complete the electronic diary entries. If a participant could not use the smartphone at a particular assessment point, no later entries were assigned.

Before the study began, all of the participants were fully informed about the content of the study and the handling of the smartphones. After they had received all of the necessary equipment, the participants were able to pursue their daily activities.

### Measures of actual physical activity

Movement and activity patterns were recorded using an accelerometer (Move I)^2^. Move I consists of a triaxial acceleration sensor with a range of ±8 g, a resolution of 12 bit and a sampling frequency of 32 Hz. Each participant wore an accelerometer on a belt around his or her chest. The recorded raw data were saved in Unisens format and transferred via a USB 2.0 interface to a computer for further analysis. The acceleration sensor can assess both movement and posture (static acceleration due to gravity). The AC (dynamic) and the DC (static) parts of the acceleration were separated by subtracting the mean value of the signal every 4 s. The AC parts of the acceleration were then used to estimate the vector magnitude of the acceleration, and the mean value of every 1 min interval was calculated. PA was quantified using the unit “milli-g.” To relate PA to the e-diary data, we computed 10-min segments of PA for the 10-min before each e-diary entry. We used 10-min segments to maintain comparability across our studies (Kanning et al., 2012; Ebner-Priemer et al., 2013). The original decision to use a 10-min segment (more details in Ebner-Priemer et al., 2013) was based on the findings of Schwerdtfeger et al. (2008), which showed promising relationships for two time frames (5, 15 min), and was based on the information that PA for a minimum of 10 min yields health benefits (Haskell et al., 2007).

### Affective states

To assess momentary affective states, we used a short scale with six items based on the Multidimensional Mood Questionnaire (MDMQ; Steyer et al., 1997), which has been explicitly developed and evaluated for use in AA (Wilhelm and Schoebi, 2007). The scale contains six items that measure the basic affective states of *valence* (unwell vs. well), *calmness* (relaxed vs. tense), and *energetic arousal* (tired vs. awake) using two bipolar items for each subscale. Homogeneity was assessed at the between-person level and the within-person level. The level-specific reliability coefficient for the between-person level was 0.92 for *valence* and 0.90 for *energetic arousal* and *calmness*. The reliability coefficient for the within-person level was 0.70 for *valence* and *calmness* and 0.77 for *energetic arousal*, resulting in satisfactory internal consistency (see Wilhelm and Schoebi, 2007 for the procedure used). The subjects indicated the extent to which they were experiencing specific affective states on a six-point scale. Answers were provided by moving a slider from the left (“0,” i.e., “discontent”) to the right (“5,” i.e., “content”) end of the bipolar scale. The scores for each subscale were obtained by summing the item scores, in which generated a range from “0” (low value) to “10” (high value). The electronic diary was provided on HTC Touch 2 smartphones that were programmed using the experience sampling software *MyExperience movisens Edition version 594* (movisens GmbH, Karlsruhe, Germany). For data management on the smartphones, the *MyExperience IDE* program *version 1.3.594* (movisens GmbH, Karlsruhe, Germany) was used.

We controlled for time of day. First, we centered the time variables. Then, we added the variables “time” and “time-square” into the analyses to control for the linear and squared effects of time.

### Analyses

The AA approach produced repeated measurements of PA and affective states (level-1) that were nested within persons (level-2). To assure chronological comparability, the accelerometers and smartphones were synchronized. We conducted separate multilevel analyses for each affect subscale using the statistical program HLM 6.0 (Raudenbush et al., 2004). We used restricted maximum likelihood estimations for the multilevel analyses. The α-level of the tests was set to *p* < 0.05.

First, we estimated the intra-class coefficient of the three subscales with unconditional models where “*y*” is not modeled as a function of another variable at level-1 or level-2. Second, we consecutively entered the predictor variables PA and time-square into each model (see Eq. 1). We analyzed the linear effect but did not find any differences between these results and the ones that we obtained when we controlled for time-square only. Thus, we deleted the linear effect. Third, we analyzed whether affective state and the last 10 min of PA before each e-diary entry (level-1) significantly varied as a function of three level-2 predictors, gender (sex), the average level of the affective state (mean_affst), and the average level of PA (mean_PA). These three level-2 predictors were inserted into each model. Thus, for each subscale, we examined the relationship between the level-1 intercept and the level-2 predictors (see Eq. 2) as well as the cross-level interaction between the level-1 slopes and level-2 predictors (see Eqs 3 and 4).

(1) $$\begin{array}{lll} \text{Level-1: }Y_{ti} & =\beta_{0i}+\beta_{1i}\;{\left(\text{PA}\right)}_{ti}+\beta_{2i}\;{\left(\text{time - square}\right)}_{ti}+r_{ti} & \end{array}$$

(2) $$\begin{array}{llll} \text{Level-2: }b_{0i} & =\gamma_{00}+\gamma_{01}\;\left(\text{sex}\right)+\gamma_{02}\;\left(\text{mean}\text{\_}\text{affst}\right) & & \\ & \;+\gamma_{03}\left(\text{mean}\text{\_}\text{PA}\right)+\mu_{0i} & \end{array}$$

(3) $$\begin{array}{llll} \text{Level-2: }b_{1i} & =\gamma_{10}+\gamma_{11}\;\left(\text{sex}\right)+\gamma_{12}\;\left(\text{mean}\text{\_}\text{affst}\right) & & \\ & \;+\gamma_{13}\left(\text{mean}\text{\_}\text{PA}\right)+\mu_{1i} & \end{array}$$

(4) $$\begin{array}{llll} \text{Level-2: }b_{2i} & =\gamma_{20}+\gamma_{21}\;\left(\text{sex}\right)+\gamma_{22}\;\left(\text{mean}\text{\_}\text{affst}\right) & & \\ & \;+\gamma_{23}\left(\text{mean}\text{\_}\text{PA}\right)+\mu_{2i} & \end{array}$$

Level-1 represents the participants’ responses (subscript *i*) on one of the basic affect subscales (*Y_ti_*) in any given diary entry (subscript *t*). *Y_ti_* is defined as the average intercept of the corresponding affect subscale across all participants (β_0*i*_) and the two level-1 predictors PA (β_1*i*_ PA*_ti_*) and time-square (β_2*i*_ time-square). These predictors are group mean-centered, with the group referring to a person (level-2). The intercepts and slopes are conceived as varying randomly. The random effect of the level-1 model is given by *r_ti_*. It is assumed to be normally distributed with a mean of “0” and a variance of σ^2^. Level-2 expresses between-subject effects. It includes the fixed effects, γ, as the average intercepts and slopes across all persons, the predictors *gender*, *mean_affst* and *mean_PA*, and the random effects *μ*_0*i*_, *μ*_1*i*_, *μ*_2*i*_, and *μ*_3*i*_. The random effects are assumed to be multivariate and normally distributed, both with expected values of “0.”

To clarify the magnitude of the effect, we standardized the effects of PA and time on the three mood subscales (see Eq. 5). The standard deviation was taken from the mean of the sample of the last 10 min of PA before each diary entry and from the sample mean for the affective states.

(5) $$\begin{array}{llll} & \text{Standardized effect} & & \\ & \beta_{1i}*\text{SD }\left(\text{activity or time - square}\right)∕ & & \\ & \;\text{SD}\left(\text{correspondent affective state}\right) & \end{array}$$

## Results

The 77 participants provided 807 data points, yielding an average of 10.5 e-diary entries per subject. The overall average level of PA across the 10-min periods prior to the measurements of the affective states was 62.0 mg/min (SD = 64.9) with a range from 12.8 to 765.2 mg/min. For the sake of comparison, note that jogging episodes equal approximately 1000 mg/min, walking episodes approximately 350 mg/min, and pure sitting episodes approximately 10 mg/min. The average levels of *valence*, *energetic arousal*, and *calmness* were 8.2, 7.0, and 8.5, respectively, representing medium to high affective states. The results for the intra-class coefficient were ρ_I_ = 0.50 for valence, ρ_I_ = 0.40 for energetic arousal, and ρ_I_ = 0.46 for calmness. This indicates that 50, 60, and 54%, respectively, of the affect subscales’ variance was caused by intra-individual variation. The distribution of PA, the time-square variable, and the three affect subscales allowed for multilevel analyses.

The random error terms of the PA slopes were not significant in the models. They were fixed because the random and fixed variability of the slopes could not be separated reliably. Nevertheless, the slopes of the last 10 min of PA before each diary entry varied between the persons but did not vary randomly as a function of the level-2 predictors gender and the average levels of the affective state and PA. The fixed and random effects on each affective state are shown in Table 1.

**Table 1:** **Fixed and random effects and variance components for valence (model 1), energetic arousal (model 2), and calmness (model 3) on actual physical activity (aPA)**.

Outcome	Fixed				Random					
Predictor	Coefficient	SE	*t*-Value (*df*)	*p*-Value	Variance estimate	SD	*X* (*df*)	*p*-Value	95-Predictive interval^a^	Slope > 0^b^
**MODEL 1: VALENCE**										
Intercept	8.199	0.123	66.41 (58)	<0.001	0.65	0.81	194.98 (57)	<0.001		
Sex	−0.297	0.350	−0.85 (58)	0.399						
VA-mean	1.094	0.076	14.42 (58)	<0.001						
aPA_mean	0.006	0.009	0.70 (58)	0.484						
aPA	0.003	0.001	2.83 (798)	0.005					−0.002; 0.007	90%
Sex	−0.007	0.003	−1.93 (798)	0.047						
V_mean	0.001	0.001	1.07 (798)	0.286						
aPA-mean	0.001	0.001	0.05 (798)	0.964						
Time	−0.002	0.002	−0.81 (61)	0.419	0.001	0.012	103.18 (60)	0.001	−0.026; 0.022	43%
**MODEL 2: ENERGETIC AROUSAL**										
Intercept	7.061	0.142	49.71 (58)	<0.001	0.875	0.935	196.38 (57)	<0.001		
Sex	−0.253	0.406	−0.62 (58)	0.536						
EA-mean	1.045	0.087	12.04 (58)	<0.001						
aPA_mean	0.012	0.010	1.14 (58)	0.261						
aPA	0.005	0.001	3.49 (798)	<0.001					0.002; 0.007	99%
Sex	−0.005	0.004	−1.21 (798)	0.229						
EA_mean	0.001	0.001	0.12 (798)	0.905						
aPA-mean	0.001	0.001	0.18 (798)	0.858						
Time	−0.024	0.003	−7.15 (61)	<0.001	0.001	0.018	127.75 (60)	<0.001	−0.059; 0.011	99%
**MODEL 3: CALMNESS**										
Intercept	8.457	0.121	69.86 (58)	<0.001	0.647	0.805	214.16 (57)	<0.001		
Sex	0.243	0.354	0.69 (58)	0.495						
C-mean	0.886	0.076	11.88 (58)	<0.001						
aPA_mean	−0.012	0.008	−1.31 (58)	0.197						
aPA	0.002	0.001	1.76 (798)	0.079					−0.0011; 0.0043	87%
Sex	−0.002	0.003	−0.47 (798)	0.641						
C_mean	−0.001	0.001	−0.66 (798)	0.512						
aPA-mean	−0.001	0.001	−1.03 (798)	0.303						
Time	0.007	0.003	2.45 (61)	0.018	0.001	0.012	142.51 (60)	<0.001	−0.0255; 0.0399	67%

*^a^Based on the assumption of normally distributed regression coefficients, the 95% predictive interval indicates the range of values between which 95% of the regression coefficients are estimated to lie (Hox, 2010). The intervals were calculated based on a model without level-2 predictors*.

*^b^Based on the assumption of normally distributed regression coefficients, this value indicates true percentage of regression coefficients that are positive (Hox, 2010). The percentages were calculated based on a model without level-2 predictors*.

### Within-person effects

*Valence* was significantly predicted by the last 10 min of PA before each diary entry (*p* = 0.005) but not by time-square (*p* = 0.419). The more our subjects were physically active during the preceding 10 min, the better and more satisfied they felt. The standardized effects were 0.07 for PA. Thus, if PA increased by 1 SD, the valence increased by 0.07 SD.

*Energetic arousal* was positively affected by the last 10 min of PA before each diary entry (*p* = 0.001) and negatively affected by time-square (*p* < 0.001). As PA during the preceding 10 min increased, the students’ sense that they were awake and energized increased. The analyses also revealed that the later it was during the day, the more tired and weak the participants felt. According to the standardized effects, when the PA or time-square figure increased by 1 SD, energetic arousal increased by 0.1 SD, and decreased by 0.3 SD, respectively.

C*almness* was not significantly predicted by the 10-min of PA preceding each diary entry (*p* = 0.079; SD = 0.04) but was predicted by time-square (*p* = 0.018; SD = 0.65). The later it was in the day, the more relaxed and calm the participants felt.

### Between-person effects

We used the level-2 predictors to investigate gender differences. In addition, we controlled for the within-person effect of PA on affective states in determining the mean level of the selected affective state and the mean level of PA. As shown in Table 1, neither the average level of the selected affective states nor the mean level of PA were significantly related to the within-person effect of PA. We found significant gender differences (*p* = 0.047) for the subscale *valence*. Women felt significantly better and more satisfied than men after they increased their activity levels.

## Discussion

To investigate the association between PA and affect in everyday life and to clarify the contradictory results of previous studies (Schwerdtfeger et al., 2008 vs. Kanning et al., 2012), we used state-of-the-art methodology: namely, AA with real-time, real-life, repeated, objective assessment. In general, the current findings support the hypothesis that there is a positive association between PA and affective states.

We documented the significant influence of PA on energetic arousal. This result confirms previously published findings that have been obtained using AA (Schwerdtfeger et al., 2008; Kanning et al., 2012).

In addition, the data revealed a significant association between PA and positive affect (*valence*). This finding is consistent with the findings of Schwerdtfeger et al. (2008) but contrary to the findings of Kanning et al. (2012).

Unlike the findings of Kanning et al. (2012), the current data analysis did not reveal a negative association between activity and *calmness*.

To explain the findings regarding the three dimensions of affect, we speculate that physiological adaptations due to short bursts of increased PA, may cause the participants to perceive themselves as having enhanced energy levels. In some studies, increased energy levels were associated with a sense of reduced calmness; however, this relationship might not necessarily hold. We speculate that individual activity thresholds (for duration and/or intensity) must be exceeded for positive affect to develop. The dimension *valence*, however, might be positively affected by even short bouts of PA.

This study communicates an important health message, especially for people who sit at work for more than 8 h per day and/or lead an inactive life. The results suggest that short and low-intensity PA, which may be conducted as brief breaks from sedentary time even in such working environments, has the potential to enhance people’s energy levels and make them feel better instantly. Accordingly, jogging intervals of half an hour are not necessary to induce such changes. It might even be speculated that everyday life activities such as stair climbing, taking a short walk around the block or doing jumping jacks may be sufficient to positively change a person’s affective state. Our use of a sample that was rather inactive at the time of the study (students during their exam period) further supports this point. However, as our participants were not chronically inactive, but rather currently inactive (because of their exams), generalization of our findings is limited regarding chronically inactive participants. We might speculate that our only currently inactive sample does profit more from small activity episodes, because of learned connections between PA and affect, compared to chronically inactive samples.

Previous findings indicated that the effects of PA were stronger when initial mood levels were low (Folkins, 1976; LaFontaine et al., 1992). However, the current study did not confirm the existence of these ceiling effects. The strength of the association between PA and *valence*/*energetic arousal* was not affected by the students’ average mood level. Therefore, even people who are already experiencing positive feelings could profit from carrying out short bursts of low-intensity activity. This finding is consistent with those of studies that have supported the assumption of a more general protective effect of PA on psychological health (Thayer, 1987; Otto and Stemmann, 1991; Gauvin and Spence, 1996; Ekkekakis et al., 2000).

Several limitations of the present study must be taken into account. First, the measurement time points were fixed. This can be problematic, if the levels of activity prior to the assessments show too little variation, decreasing the power of the assessment approach. However, despite this disadvantage, we showed significant associations between activity and two of the three affective dimensions. To overcome the limited variation in the PA conducted directly before the e-diary assessments, interactive AA has been recommended (Ebner-Priemer et al., 2013). In interactive AA, e-dairy entries are triggered not by time but by the variables of interest, such as activity. Second, only university students were questioned, leaving out less educated or older people with a lower socio-economic status. Third, the assessment period covered only 1 day. Fourth, we primarily focused on the association between PA and affective states. Future studies should further analyze the association between PA and the degree of a subsequent change in affective states over a longer period of time. This search for an effective “*intensity threshold*” could aid in the development of more precise recommendations regarding activities that could interrupt sedentary behavior.

The key message of the current study is that repeated short periods of everyday life activities have the potential to positively influence affective states and subjective well-being and, in the long term, support a healthy lifestyle.

## Conflict of Interest Statement

The authors declare that the research was conducted in the absence of any commercial or financial relationships that could be construed as a potential conflict of interest.

## Footnotes

^1^www.ambulatory-assessment.org

^2^www.movisens.com
